# Supplementary material for: Agreement between upper and lower limb measures to identify older adults with low skeletal muscle strength, muscle mass and muscle quality
Source: PLoS One. 2022 Jan 21;17(1):e0262732. doi: 10.1371/journal.pone.0262732 (PMC8782376; doi:10.1371/journal.pone.0262732)
Supplement: Attachment — Submitted filename: Response letter - PLOS ONE.docx [file pone.0262732.s001.docx]

Eduardo Caldas Costa, PhD

Department of Physical Education, Federal University of Rio Grande do Norte

Campus Universitário BR 101, Natal 59072-790, Brazil

Phone/Fax: +55 84 3215 3455

E-mail: [ecc@ufrnet.br](mailto:ecc@ufrnet.br)

November 04^th^, 2021

To Dr. Emily Chenette

Editor-in-Chief
**PLOS ONE**

RE: Response letter of the manuscript ID PONE-D-21-18967, entitled ‘**Agreement between upper and lower limb measures to identify older adults with low skeletal muscle strength, muscle mass and muscle quality**’, for review.

Dear Dr. Emily Chenette (Editor-In-Chief)

We appreciate the consideration of our manuscript for review in the PLOS ONE, which is a cutting-edge journal in the field of biomedical literature. We have read with great interest the concerns addressed by the reviewers and we are thankful for the time they have spent reviewing our manuscript. All suggestions were valuable to the improvement of the manuscript and they made the authors rethink many aspects of the tackled issues. Therefore, that said, we listed and respond to all concerns and issues raised by the reviewers and editors in our manuscript. The changes are listed below and highlight in the manuscript:

**Editor and Reviewer Comments**

**Editor comments**

**Comment 1:** The premises and the aim of the study are interesting given the importance of detecting persons at risk for/affected by sarcopenia.

**Response:** Thank you for the feedback and comments. We are grateful for the time you spent reviewing our manuscript.

**Comment 2:** In my opinion the major limitation of the study is the fact that lower limbs muscle strength was measured by means of the 30-s chair stand test rather than with a dynamometer. As Authors state 30-s chair stand test assesses the performance on a functional test, being not only an indicator of muscle strength.

**Response:** Thank you for the feedback and the opportunity to clarify this point. Our rationale was based on the guideline from the European Working Group on Sarcopenia in Older People (EWGSOP2), which recommends assessing skeletal muscle strength by using interchangeable tests; i.e. handgrip strength test or chair stand test. This guideline, which is the most used in clinical practice worldwide and designed “to increase consistency of research design, clinical diagnoses and ultimately, care for people with sarcopenia” states that:

*“The chair stand test (also called chair rise test) can be used as a proxy for strength of leg muscles (quadriceps muscle group). The chair stand test measures the amount of time needed for a patient to rise five times from a seated position without using his or her arms; the timed chair stand test is a variation that counts how many times a patient can rise and sit in the chair over a 30-second interval [64, 67, 68]. Since the chair stand test requires both strength and endurance, this test is a qualified but convenient measure of strength”. (pg. 20).*

Therefore, although we fully agree that handgrip strength test and chair stand test are different, their use are recommended by the most used sarcopenia guideline in clinical practice. Therefore, our findings might be useful to rethink the interchangeable use of these different tests in the practical algorithm for sarcopenia case-finding, diagnosis and severity.

**Comment 3:** It would be interesting to add in Results section and/or in Table 1 other general characteristics of the sample, as indicators of physical performance, subjects' level of physical activity.

**Response:** Thank for this feedback. Information about participants’ physical activity level has been added accordingly.

**Methods**

***2.7 Physical activity***

Physical activity level was determined by the Brazilian version of the Minnesota Leisure Time Activities Questionnaire for older adults. The physical activities were classified as light, moderate or vigorous considering the absolute intensity (metabolic equivalents; METs) for each specific age (40-64 years; ≥ 65 years), based on the American College of Sports Medicine. Participants who performed ≥600 MET/min/wk of moderate-vigorous physical activities were considered as ‘active’, while those who performed < 600 MET/min/wk were considered as ‘inactive’.

**Results**

Additionally, 58.4% (n = 225) were physically active and 41.6% (n = 160) were physically inactive.

**Comment 4:** Finally, the Authors should discuss the reason why the agreement between upper and lower limbs muscle strength and quality is so relevant.

**Response:** Thank you for the feedback. In the discussion section, this information has been included accordingly.

From a clinical perspective, our findings might be useful to rethink the recommendation of the interchangeable use of the HGS and CST in the EWGSOP2 (7) practical algorithm for dynapenia and sarcopenia case-finding, diagnosis and severity, mainly due the limited agreement between these UL and LL tests to identify low SMS and SMQ in older adults. Based on our findings, using the HGS an older adult can be classified as ‘normal SMS’ and nonsarcopenic while using CST his/her classification can be dynapenia (low SMS) or even sarcopenia. The opposite scenario is also possible; i.e. ‘normal SMS’ and nonsarcopenic using CST and dynapenia or sarcopenia using HGS. Thus, misinterpretation regarding the clinical identification of dynapenia and sarcopenia can occur, which can favor unappropriated interventions delivered for these individuals.

**Comment 5:** I suggest a punctuation check (see Results section, lines 167-168: change 35,3% in 35.3%; and 32,99% in 32.99%).

**Response:** Thank you for the feedback. The changes have been made accordingly.

**Reviewer #1:** The authors assess the agreement between upper and lower limb measures of muscle mass, strength and quality in older adults. The topic is interesting, the sample size is adequate and statistical analysis is well conducted. The paper is clearly written.

**Response:** Thank you for the feedback and comments. We are grateful for the time you spent reviewing our manuscript.

Sincerely,

Eduardo Caldas Costa, PhD
